# Telomere Length as Both Cause and Consequence in Type 1 Diabetes: Evidence from Bidirectional Mendelian Randomization

**DOI:** 10.3390/biomedicines13040774

**Published:** 2025-03-22

**Authors:** Guanping Wei, Ruiping Chen, Shupeng Liu, Shenhua Cai, Zhijun Feng

**Affiliations:** 1 Department of Emergency, The First Affiliated Hospital of Sun Yat-sen University, Guangzhou 510080, China; weigp3@mail.sysu.edu.cn; 2 Department of Thoracic Surgery, The First Affiliated Hospital of Sun Yat-sen University, Guangzhou 510080, China; chenrp8@mail.sysu.edu.cn; 3 Guangdong Provincial Key Laboratory of Tropical Disease Research, Department of Radiation Medicine, School of Public Health, Southern Medical University, Guangzhou 510515, China; 20220062@smu.edu.cn; 4 Department of Breast Surgery, The First Affiliated Hospital of Sun Yat-sen University, Guangzhou 510080, China

**Keywords:** telomere length, diabetes, diabetic complication, Mendelian randomization

## Abstract

**Background/Objectives:** Diabetes is the most prevalent metabolic disease globally, characterized by dysregulated glucose control and accompanied by multiple refractory complications. As a critical marker of cellular homeostasis, telomere length (TL) may be associated with the progression of diabetes. However, the causal relationship between diabetes and TL remains unclear, particularly whether cellular homeostasis imbalance acts as a consequence of diabetic complications or a precipitating factor in disease development. **Methods:** We performed a bidirectional Mendelian randomization (MR) analysis using genome-wide association study (GWAS) data. Following the three core assumptions of MR analysis, we conducted quality control on all instrumental variables to ensure methodological rigor. The inverse variance weighted (IVW) method served as the primary analytical method, supplemented by additional MR methods to evaluate the significance of the results. Furthermore, we performed sensitivity analyses to ensure the reliability and robustness of the findings. **Results:** Forward analysis revealed that shortened TL significantly increases the risk of broadly defined Type 1 diabetes (T1D) and unspecified types of diabetes (*p* < 0.05). Additionally, we identified a positive causal relationship between TL and several diabetes-related complications, including co-morbidities, diabetic nephropathy, and diabetic ketoacidosis (*p* < 0.05). Interestingly, the reverse analysis demonstrated a positive causal effect of T1D and its complications on TL (*p* < 0.05); however, this effect disappeared after adjusting for insulin use (*p* > 0.05). **Conclusions:** Bidirectional MR analyses revealed a complex relationship between TL and T1D, where shortened telomeres increase T1D risk while T1D itself may trigger compensatory mechanisms affecting telomere maintenance, with insulin playing a crucial regulatory role in this relationship. These findings suggest telomere biology may be fundamentally involved in T1D pathogenesis and could inform future therapeutic approaches.

## 1. Introduction

Diabetes, characterized by elevated blood glucose levels, is a chronic metabolic disease and a significant global public health challenge [1,2,3]. Diabetes is mainly classified into type 1 diabetes (T1D), type 2 diabetes (T2D), gestational diabetes, and unspecified diabetes [4]. T1D results from the autoimmune destruction of pancreatic β-cells, leading to insufficient insulin secretion [5,6], whereas T2D is primarily due to insulin resistance and β-cell dysfunction [7,8]. Both types of diabetes can cause severe complications [9], including cardiovascular diseases, nephropathy, retinopathy, and neuropathy, which significantly increase the disease burden and mortality risk for diabetic patients. According to the International Diabetes Federation (IDF), approximately 537 million adults worldwide had diabetes in 2021, and this number is projected to rise to 643 million by 2030 [10,11]. Diabetes and its complications not only severely impact patients’ quality of life, but also impose a substantial economic burden on healthcare systems globally. Therefore, understanding the pathological mechanisms of diabetes, especially its relationship with biological aging-related factors, is crucial for disease prevention and management.

Telomeres, protective structures located at the ends of chromosomes, consist of repetitive DNA sequences (TTAGGG) [12,13]. Telomere length (TL) shortens with each cell division until reaching a critical length, leading to cellular senescence or apoptosis [14,15]. Consequently, TL is widely considered a biomarker of cellular biological age [16]. Numerous studies have shown that shortened TLs are associated with various age-related diseases, such as cardiovascular diseases [17,18], cancer [19,20], and nerve degenerative diseases [21,22,23]. TL reflects not only cellular division history and biological age, but is also influenced by genetic and environmental factors. For instance, oxidative stress and chronic inflammation are major environmental factors accelerating TL shortening [24]. Additionally, certain genetic variations are associated with changes in TL, providing unique opportunities to study the causal relationship between TL and diseases. Although existing research indicates a link between TL and diabetes and its complications [25,26,27], the causal direction remains unclear. Shorter TL might increase diabetes risk [28,29], while diabetes, as a common metabolic syndrome, might affect TL by altering metabolic states and inflammation levels [30,31,32]. Given the prevalence of diabetes and its complications, exploring the causal relationship between TL and diabetes is essential.

Mendelian randomization (MR) is a method that uses genetic variations to infer causal relationships [33,34]. Genetic variations are randomly assigned at conception and are not influenced by environmental factors, allowing them to serve as “natural experiments” to explore causal relationships between exposures and diseases. MR uses genetic variants (typically single nucleotide polymorphisms, SNPs) as instrumental variables (IVs) to avoid confounding and reverse-causation issues common in traditional observational studies, thus providing more reliable causal evidence [35,36]. Bidirectional MR analysis extends the application by simultaneously evaluating the bidirectional causal relationship between two variables. This method is particularly suitable for investigating the complex interactions between TL and diabetes and its complications. Utilizing genome-wide association study (GWAS) data, bidirectional MR can reveal potential bidirectional causal relationships between TL and diabetes, offering new insights into the biological mechanisms of the disease.

This study investigates the bidirectional causal relationship between TL and diabetes utilizing GWAS data. Specifically, we examine whether TL alterations causally influence diabetes risk and its complications, and conversely, whether diabetes and its complications exert causal effects on TL. By examining the regulatory influence of genetic determinants in these interactions, our findings provide scientific evidence that may inform both preventive strategies and therapeutic approaches for diabetes management.

## 2. Materials and Methods

### 2.1. Data Sources

**Figure 1:**
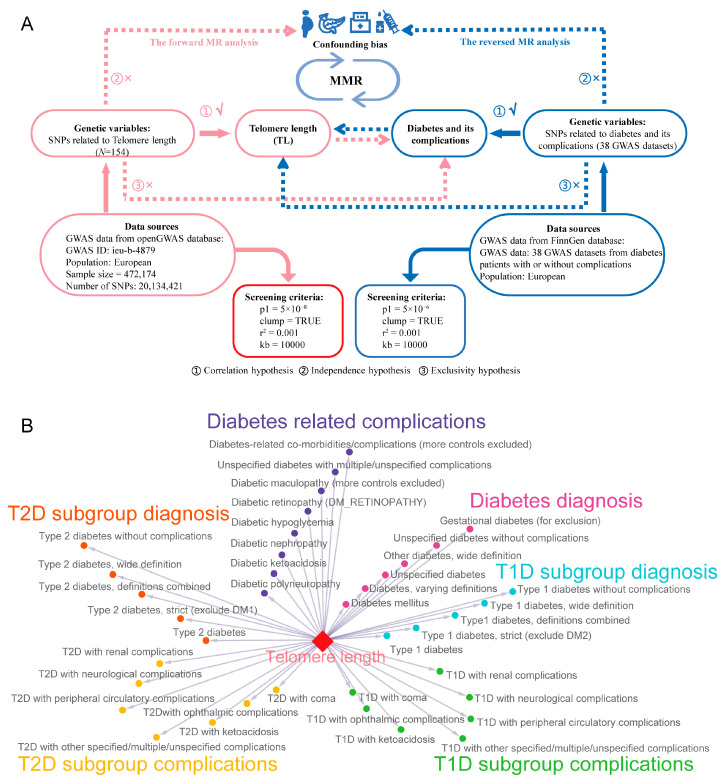
Flowchart of the study design and the distribution of the main datasets in forward Mendelian randomization (MR) analysis. (**A**) Study design; red section in A represents forward MR analysis; blue section in A represents inverse MR analysis; “X” represents exclusion; “√” represents inclusion; ① ② ③ represent three core hypotheses of MR analysis; the symbols in “Confounder bias” part represent BMI, pancreatic disease, lipid profiles, and insulin use in sequence. (**B**) The distribution of the main datasets; red diamond represents telomere length (TL) as a exposure; arrows indicate the direction of MR analysis, and the end points to the outcome; different dot colors represent different outcome classifications. GWAS, genome-wide association study; MMR, multivariate MR analysis; T1D, type 1 diabetes; T2D, type 2 diabetes.

In this study, GWAS summary data related to TL were obtained from the openGWAS database (https://gwas.mrcieu.ac.uk/, accessed on 20 December 2024) [37]. GWAS summary data for diabetes and its complications were primarily sourced from the FinnGen database (https://risteys.finngen.fi/, accessed on 20 December 2024) [38], comprising 38 different GWAS datasets (Table S1). In this study, we employed bidirectional two-sample MR as our primary analytical approach (Figure 1A), with the forward MR analysis defining the direction between TL and diabetes and its complications, and the reverse MR analysis defining the opposite (Figure 1A). This MR analysis investigated causal relationships at a general level, and performed subgroup analyses based on disease type (Figure 1B), including overall diabetes diagnosis-related MR analysis (6 datasets), T1D subgroup (5 datasets), and T2D subgroup (5 datasets), as well as overall diabetes complications (8 datasets), the T1D-related complications subgroup (7 datasets), and the T2D-related complications subgroup (7 datasets) MR analyses (Figure 1B). The datasets utilized in this study were sourced from European populations, with preference given to the largest sample size or most case-rich datasets when multiple options were available. This study is reported using the Strengthening the Reporting of Observational Studies in Epidemiology Using Mendelian Randomisation (STROBE-MR) guidelines [39].

### 2.2. IVs Selection

#### 2.2.1. Identifying Exposure-Related IVs

We used the “TwoSampleMR” (version 0.6.8) R package to identify SNPs closely related to the exposure (telomere length, TL) and selected them as IVs [40]. For forward analysis, IVs were obtained with the parameters *p* = 5 × 10^−8^, r^2^ = 0.001, and kb = 10,000 [41,42]. For reverse analysis, due to the insufficient number of SNPs in multiple diabetes complication datasets, the *p*-value threshold for IVs was adjusted to *p* = 5 × 10^−6^ (Figure 1). Other parameters remained unchanged from the forward analysis. IVs were selected to meet the three core hypotheses of MR analysis, as follows [36,43]: ① the correlation hypothesis, where SNPs are strongly associated with the exposure; ② the independence hypothesis, where SNPs are independent of confounders; ③ the exclusivity hypothesis, where SNPs affect the outcome only through the exposure (Figure 1A). To ensure IV validity, we conducted preliminary quality checks, focusing on the availability of sample size and effect allele frequency (EAF) data. If sample size data were missing, we supplemented them using relevant database information; missing EAF data were filled using the “1000 Genomes Project” data [44]. This process ensured the accuracy and completeness of the selected IVs.

#### 2.2.2. Excluding Confounders

To exclude potential confounding IVs, we used the “LDtrait” Tools from the “LDlink” database (https://ldlink.nih.gov/?tab=ldtrait, accessed on 20 December 2024) to identify potential pleiotropic effects [45]. We specifically screened for associations with phenotypic data related to BMI (in forward analysis), pancreatic diseases (in forward analysis), diabetes (in forward analysis), or TL, and insulin utilization (in reverse analysis). To further ensure the IVs’ strength, we calculated the F-statistics for all exposure-related IVs using the formula F = beta^2^/se^2^. We implemented a minimum F-value threshold of 10, which is widely recognized in the MR literature as indicative of a sufficient IVs strength to avoid weak IVs bias [46,47,48]. This threshold helps minimize the potential bias toward the observational association that can occur with weaker IVs. Only IVs with F-values exceeding this threshold were included in our final analysis, thereby confirming the IVs’ validity and enhancing the overall accuracy and reliability of our causal estimates.

#### 2.2.3. Identifying Outcome-Related IVs

Based on exposure-related IVs, we used the “TwoSampleMR” R package to extract IVs related to the study outcomes, with parameters rsq = 0.8 and maf_threshold = 0.3 [41,42]. Subsequently, we integrated exposure- and outcome-related IVs, ensuring the matching of effect alleles and reference alleles, and used “MR-PRESSO” (version 1.0) combined with the “RadialMR” (version 1.1) package to identify and remove outlier IVs [49,50]. This data cleaning process yielded the final list of IVs for MR analysis.

### 2.3. MR Analysis

MR analysis was performed using the “TwoSampleMR” R package for two-sample MR analysis [51]. Both forward and reverse analyses used the same MR methods, with inverse-variance weighting (IVW) as the primary method [52,53], supplemented by MR Egger [54], weighted median [55], simple mode [56], and weighted mode methods [57]. Significant results were defined as those with a *p*-value under 0.05 in the IVW method, and beta values consistent with IVW in other methods [58]. We obtained odds ratios (ORs) and 95% confidence intervals (CIs) for each analysis direction using the “TwoSampleMR” R package (version 0.6.8) to assess the causal effects of MR analysis. In this exploratory study, we report unadjusted *p* values despite performing multiple comparisons to maintain analytical sensitivity. We acknowledge this methodological limitation and recognize that the resulting findings would require validation in future studies [59].

### 2.4. Multivariable MR Analysis

Given the prevalence of diabetes as a metabolic disorder and the widespread use of insulin as a treatment modality, our study employed a multivariate MR analysis to account for insulin product utilization, assess the independent causal relationship between diabetes and TL, and elucidate the potential influence of insulin use on TL. The findings of the multivariate MR analysis were conducted using the “TwoSampleMR” R package [60], with the IVW method as the primary analytical approach. A *p*-value of less than 0.05 was considered statistically significant [61].

### 2.5. Sensitivity Analysis

A sensitivity analysis was conducted to evaluate the presence of horizontal pleiotropy and heterogeneity among the included IVs [62]. Heterogeneity was identified through the Cochran’s Q test, with a significance level of *p* < 0.05 indicating the presence of heterogeneity [34]. In instances where heterogeneity was detected, the random-effects model–IVW method was employed to reassess the results of the MR analysis. Horizontal pleiotropy was evaluated through the implementation of the “MR Egger intercept” method with statistical significance determined by *p*-values below 0.05 [63]. Sensitivity analyses were depicted using scatter plots and funnel plots. Additionally, single-SNP effect estimation and leave-one-out analysis were performed to assess the individual influence of each SNP on the results of the MR analysis.

## 3. Results

### 3.1. Forward Analysis Shows Different Causal Effects of TL on Diabetes Types and Its Complications

**Figure 2:**
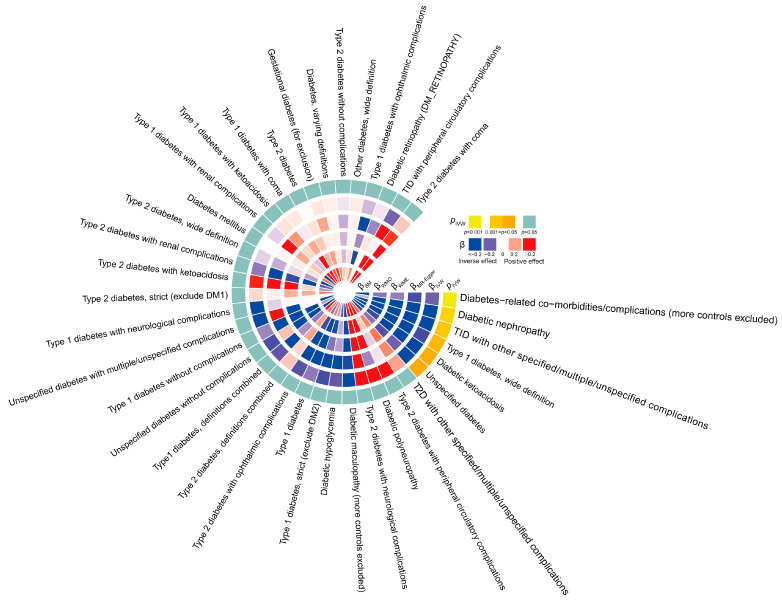
The heatmap displays all results from forward MR analysis. The first five circles, from inside to outside, represent the estimated effects (β values) calculated using five methods: simple mode (βSM), weighted mode (βWMO), weighted median (βWME), MR Egger (βMR Egger), and inverse variance weighting (βIVW). White indicates β equals 0, blue represents negative β values (with dark blue for β < −0.2), and red shows positive β values (with dark red for β > 0.2). The outermost circle represents the *p*-values corresponding to the IVW method: light green indicates *p* < 0.05, dark yellow represents *p* values between 0.001 and 0.05, and yellow indicates *p* < 0.001.

We conducted a screening of 154 SNPs closely associated with TL, with corresponding F values listed in Table S2. Following the exclusion of confounders and implementation of quality control measures, such as identifying and removing outliers, a final set of SNPs for MR analysis was obtained. Utilizing the IVW method as the primary MR approach, supported by four additional methods, the analysis results (Figure 2) indicate that TL had a reverse causal effect on T1D (wide definition) (IVW β = −0.173, IVW *p* = 0.038), undefined diabetes (IVW β = −0.209, IVW *p* = 0.045), diabetes-related co-morbidities/complications (IVW β = −0.139, IVW *p* < 0.001), diabetic nephropathy (IVW β = −0.242, IVW *p* = 0.022), diabetic ketoacidosis (IVW β = −0.196, IVW *p* = 0.043), and other T1D-related complications (IVW β = −0.248, IVW *p* = 0.029). No significant effects were observed in other analysis directions such as TL and gestational diabetes, or TL and T2D and its complications (*p* > 0.05, Figure 2). Detailed results of the five MR methods for each analysis direction are provided in Table S3. These findings suggest that shorter TL increases the risk of T1D, specific types of diabetes, and some serious and harmful diabetes complications.

### 3.2. Sensitivity Analysis in Forward Analysis

**Figure 3:**
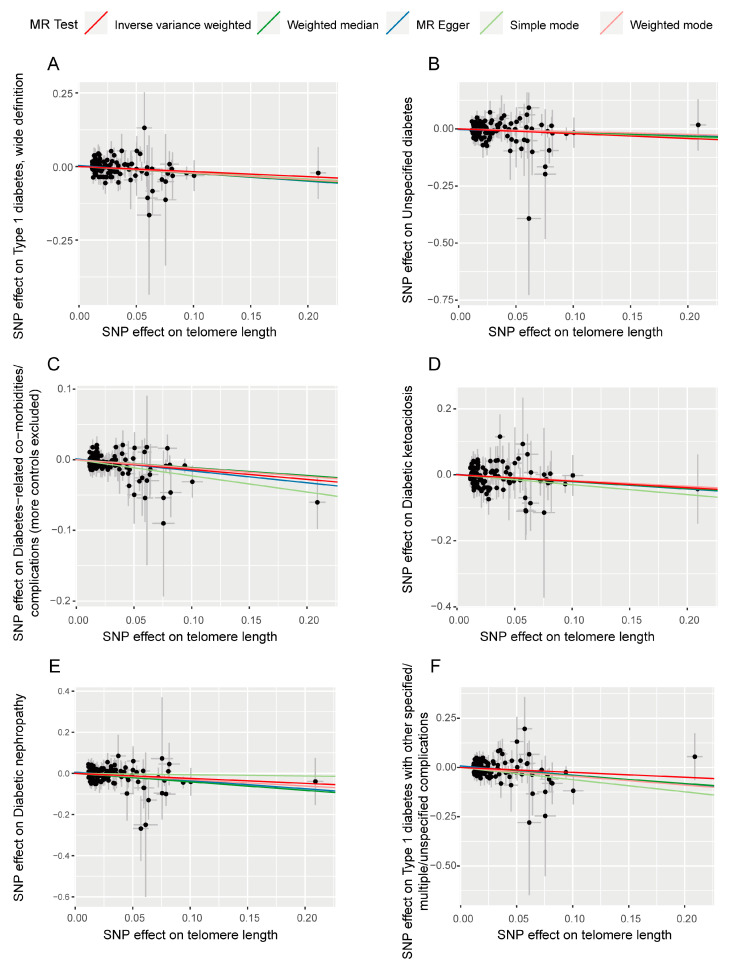
Scatter plots demonstrating the causal effect relationships. (**A**–**F**) represent analyses using telomere length as exposure with different diabetes outcomes: (**A**), type 1 diabetes; (**B**), undefined diabetes; (**C**), diabetes complications, (**D**), diabetic ketoacidosis; (**E**), diabetic nephropathy; (**F**), unspecified complications of Type 1 diabetes. The horizontal axis represents the effects of instrumental variables (IVs) on exposure, while the vertical axis shows the effects of IVs on outcomes. Each colored line represents the linear fitting trend obtained through different Mendelian randomization (MR) analysis methods. The dots represent individual single-nucleotide polymorphisms (SNPs) used as IVs in the analysis.

**Figure 4:**
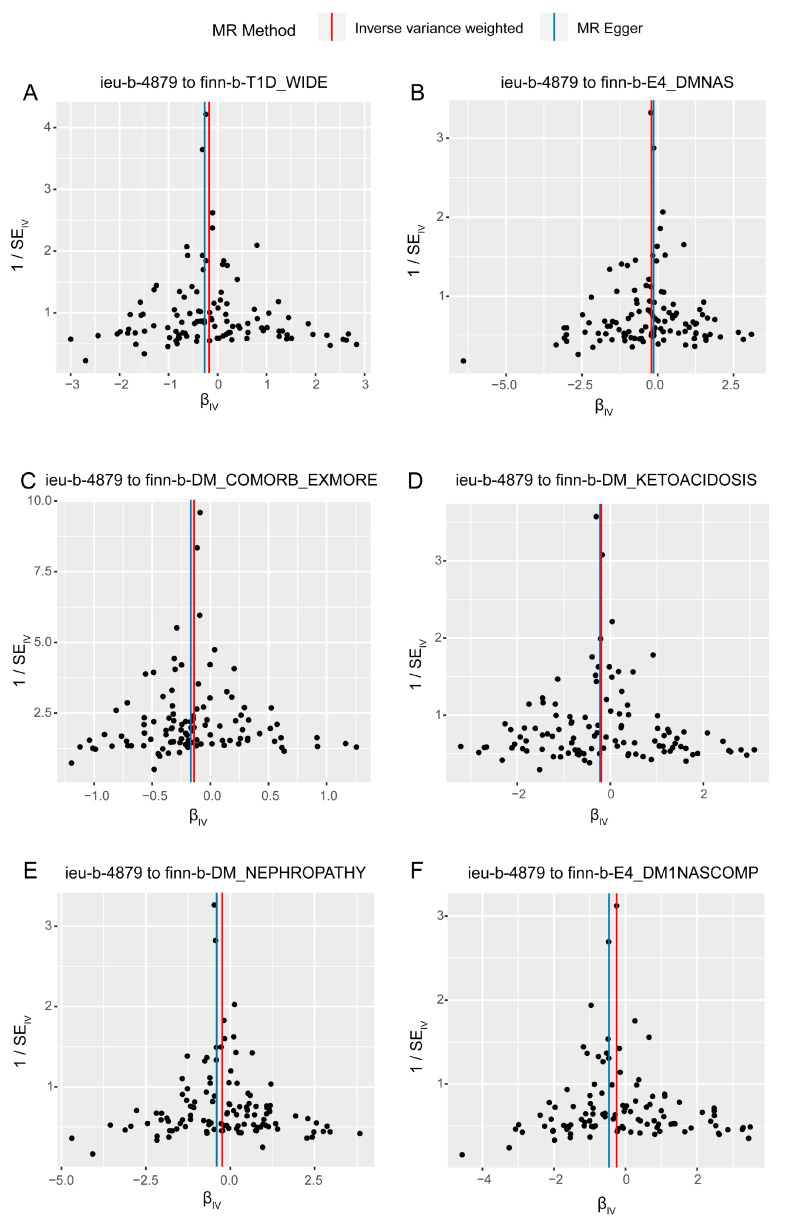
Funnel plots illustrating the distribution of causal effect from single-nucleotide polymorphisms (SNPs). (**A**–**F**) represent analyses using telomere length as exposure with different diabetes outcomes: (**A**), type 1 diabetes; (**B**), undefined diabetes; (**C**), diabetes complications, (**D**), diabetic ketoacidosis; (**E**), diabetic nephropathy; (**F**), unspecified complications of Type 1 diabetes. The horizontal axis represents the effects of instrumental variables (IVs) in the MR analysis, while the vertical axis represents the inverse of the standard error (1/SE). Colored lines show the linear fitting trends between the vertical and horizontal axes using different Mendelian randomization (MR) analysis methods. Each dot represents an individual SNP used as an IV in the analysis.

According to the forward analysis, pleiotropy and heterogeneity tests were conducted in each direction, but there was no statistical significance in either direction (Table S4). This indicates the reliability and robustness of the current analysis results. Additionally, single SNP effect estimates (Figure S1) and leave-one-out analysis (Figure S2) for the six significant causal directions confirm the robustness of the results. Single SNP effect estimates show that each SNP’s contribution to the causal relationship between TL and diabetes and its complications was relatively independent and consistent. Leave-one-out analysis demonstrated that removing any single SNP did not significantly affect the overall results, indicating no single SNP dominated the analysis. The scatter plot (Figure 3) and funnel plots (Figure 4) also show no significant abnormal SNP distributions in significant analysis directions.

### 3.3. Reverse Analysis Confirms Different Causal Effects of Diabetes Types and Complications on TL

To further confirm the causal effects of different types of diabetes and their complications on TL, we conducted reverse MR analysis. This analysis yielded six significant results (*p* < 0.05, Table 1), showing that T1D (definitions combined) (IVW β = 0.006, IVW *p* < 0.001), T1D (strict, excluding DM2) (IVW β = 0.006, IVW *p* < 0.001), T1D without complications (IVW β = 0.005, IVW *p* < 0.001), T1D with coma (IVW β = 0.003, IVW *p* = 0.025), and diabetic hypoglycemia (IVW β = 0.006, IVW *p* = 0.042) had positive causal effects on TL, while diabetic retinopathy (IVW β = 0.011, IVW *p* = 0.042) had an inverse causal effect on TL (Table 1). However, T2D and its related complications showed no significant causal effects on TL. Detailed results of the five MR methods for each analysis direction are provided in Table S5. In the reverse analysis, the sensitivity analysis results show no pleiotropy or heterogeneity (*p* > 0.05, Table S6). These findings indicate that T1D might have a more direct impact on TL changes, while the relationship between T2D and TL might be influenced by more uncontrolled confounders. Therefore, TL might serve as an important biomarker in predicting and intervening in T1D, offering new insights into the biological nature of this disease.

Given the widespread clinical use of insulin products among individuals with diabetes, a multivariate MR analysis was conducted to identify potential bias from treatment factors, specifically the use of insulin products, in the reverse analysis. According to the results, once insulin products were adjusted for, all six dimensions of the MR analysis were non-significant (Table 1). This phenomenon suggests that insulin therapy may serve as an important effect modifier mediating the phenotypic association between diabetes and TL. Additionally, the directionality of association remained consistent before and after adjustment, suggesting that insulin therapy may attenuate rather than reverse the impact of diabetes on telomere dynamics. These findings highlight the potential biological interactions between insulin signaling pathways and telomere maintenance mechanisms, which may have important implications for understanding how diabetes management strategies influence cellular aging processes.

**Figure 5:**
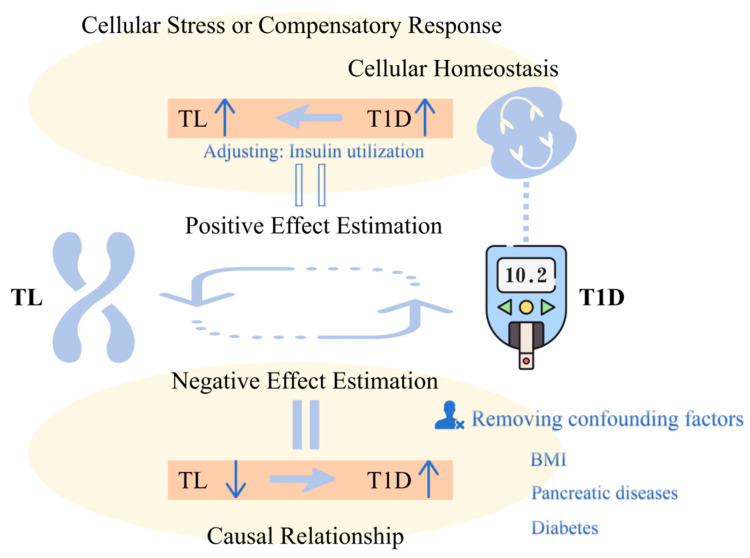
Bidirectional causal relationship model between telomere length (TL) and type 1 diabetes (T1D). The bidirectional causal relationship between TL and T1D is represented by a circular arrow. The equal signs indicate the positive and negative causal effects obtained from Mendelian randomization analysis in the current study.

Based on the evidence, we propose a hypothesis of a bidirectional regulatory relationship between TL and T1D. On one hand, the abnormal shortening of telomeres may increase the risk of T1D development, which is consistent with the clinical feature that T1D is commonly observed in young populations. On the other hand, when T1D occurs, metabolic dysregulation triggers a series of compensatory protective mechanisms, including the upregulation of telomerase activity, the enhancement of DNA damage repair, the activation of antioxidant defense systems, and the remodeling of autophagy pathways, collectively maintaining telomere homeostasis. This compensatory mechanism explains the positive causal effect of T1D and some of its complications on TL that we observed. Figure 5 illustrates this bidirectional regulatory relationship model. Furthermore, the disappearance of this causal effect after insulin therapy further confirms the central role of insulin in maintaining TL and regulating cellular homeostasis.

## 4. Discussion

This study has employed bidirectional MR to investigate the causal relationship between TL and diabetes mellitus and its complications. Our forward analysis reveals that shortened TL significantly increased the risk of T1D, undefined diabetes, and several diabetes-related complications, including diabetic nephropathy, diabetic ketoacidosis, and other T1D-related complications. Notably, no significant causal effects were observed between TL and T2D or gestational diabetes, suggesting heterogeneity in TL’s impact across different diabetes types. Sensitivity analyses, including single-SNP effect estimates and leave-one-out analyses, confirmed the robustness of these findings. Intriguingly, our reverse MR analysis demonstrated the positive causal effects of T1D, T1D without complications, T1D with coma, and diabetic hypoglycemia on TL, while diabetic retinopathy exhibited an inverse effect. This bidirectional relationship suggests complex interactions between TL and diabetes pathophysiology. Importantly, after adjusting for insulin therapy, all significant associations disappeared, indicating insulin’s crucial role in modulating the relationship between diabetes and telomere biology. These findings suggest that telomere dynamics may be intricately involved in T1D pathogenic processes and could potentially serve as both a risk indicator and a marker of disease progression. Furthermore, our results highlight the distinct biological mechanisms underlying different diabetes subtypes, and emphasize the importance of considering telomere biology in understanding the etiology and progression of T1D.

TL is a critical indicator of cellular activity, closely related to cellular proliferation capacity. As TL shortens, cellular proliferation and differentiation capacity gradually decline [64,65]. When TL shortens beyond a certain threshold, cells cease proliferation and differentiation, becoming senescent [66,67,68]. This analysis found a negative causal relationship between TL and specific types of diabetes, suggesting that abnormalities in cellular proliferation and differentiation capacity might play a crucial role in the pathogenesis of specific diabetes types [69,70]. For example, from a clinical perspective, T1D onset is not significantly influenced by factors such as obesity and advanced age [71], and it is more common in young patients with relatively long TLs. Based on this analysis, shortened TL might be an important trigger for T1D onset in young patients. Shortened TL can cause proliferation and differentiation disorders in pancreatic secretory cells [16,72], activating the body’s immune surveillance system and damaging pancreatic acinar cells, leading to β-cell dysfunction, reduced insulin production, and ultimately the development of T1D. Similarly, the negative causal relationship between TL and diabetes complications, especially diabetic ketoacidosis and diabetic nephropathy, can be explained by changes in cellular proliferation and differentiation capacity. Telomere shortening might lead to a functional decline in critical tissues like the kidneys, accelerating complication development. As these organs’ cells with shorter TL cannot effectively regenerate and repair, organ function gradually declines, exacerbating long-term diabetes damage. In this theoretical framework, TL is not only a marker of aging, but also a key regulator of cellular health and disease progression. Therefore, protecting telomeres and delaying their shortening might be a new therapeutic target for specific types of diabetes and severe complications [25,73]. Enhancing telomerase activity or using gene editing technologies to maintain telomere stability could improve cellular activity and regeneration at the molecular level, slowing disease progression and improving clinical symptoms and quality of life for diabetes patients.

Diabetes, as a major manifestation of metabolic syndrome, causes significant changes in the body’s metabolic state and system functions [74,75]. The key treatment goal is to maintain blood glucose stability and protect organ functions through various interventions to prevent complications. From this perspective, we assessed the causal effect of diabetes on TL. Without considering treatment factors, diabetes had a positive causal effect on TL, which can be seen as the body’s negative feedback mechanism [76]. Telomere shortening might trigger diabetes onset, and once the disease occurs, the body might activate other pathways to prevent further telomere shortening, aligning with general cell lifecycle patterns. From the perspective of cellular proliferation and differentiation capacity, this negative feedback mechanism might extend TL to maintain cellular self-renewal [77,78] and repair capacity [79,80], protecting cells from further damage [81]. This mechanism is particularly important in diabetes, as it helps mitigate tissue damage caused by metabolic abnormalities. However, in a diabetes-induced metabolic disorder environment, the positive regulation of TL might not always be beneficial [29,82]. Although this regulation might help maintain cell function to some extent, it could increase the risk of abnormal cellular changes in the long term. These abnormal changes might involve cell cycle control disorders and apoptosis inhibition, leading to cellular dysfunction and increased tumor risk. Therefore, understanding and regulating telomere biology in diabetes might require a balanced approach. While extending telomeres might provide short-term cell protection, long-term strategies should focus on restoring and maintaining metabolic stability and ensuring normal cellular proliferation and differentiation regulation. In this analysis, reverse MR analysis results confirm that diabetes might promote telomere extension through this negative feedback pathway, partially counteracting disease-induced cellular differentiation and proliferation disorders. These findings highlight the complexity of diabetes treatment strategies and the value of TL as a potential biomarker in disease management. Blood glucose-lowering treatment is central to diabetes management. In our study, we included insulin product usage in reverse MR analysis and conducted multivariable MR analysis, finding that the positive causal effect of diabetes on TL became insignificant after considering insulin usage. This result suggests that insulin might stabilize TL by regulating cellular metabolic homeostasis [83,84]. Understanding these mechanisms is crucial for comprehending diabetes’s biological basis. Telomeres, DNA–protein complexes at chromosome ends, primarily protect chromosomes from damage [85]. As cells divide, telomeres gradually shorten, eventually leading to cell division cessation and senescence [86]. In diabetic patients, sustained high-glucose environments accelerate metabolic disorder, increasing cellular aging and dysfunction risk. Insulin treatment’s potential mechanisms in maintaining TL stability include the following: ① Improving blood glucose control and reducing oxidative stress, possibly slowing telomere shortening [87,88]. ② Directly or indirectly influencing telomerase activity, a reverse transcriptase that can extend telomeres [89,90]. Maintaining telomerase activity helps stabilize telomeres, counteracting the negative feedback regulation of TL post-diabetes onset. ③ Regulating metabolic homeostasis in diabetic patients, reducing metabolic syndrome symptoms, and improving intracellular environments, thus helping stabilize TL [91,92]. These results emphasize insulin’s traditional role in blood glucose control, and reveal its potential biological effects in regulating cellular aging and telomere stability. Understanding these mechanisms can inform new diabetes treatment strategies, potentially involving new drugs or therapies targeting telomere maintenance to delay cellular aging and improve long-term health for diabetes patients.

MR’s core advantage is overcoming confounding and reverse causation issues in traditional observational studies. Selecting appropriate IVs is crucial for effective and reliable MR analysis [35,36]. In this study, we selected genetic variants strongly associated with TL and various types of diabetes and their complications from large genomic databases, ensuring IV independence and the absence of pleiotropy. We also calculated F-statistics to assess IV strength, ensuring the genetic variants used provided sufficient statistical power for reliable causal inference. In practice, we selected disease populations as outcomes different from the exposure population, avoiding sample overlap bias. To validate the MR analysis’s robustness, we conducted sensitivity analyses and combined different MR methods to comprehensively evaluate the causal estimates’ validity, enhancing result robustness. Using MR Egger regression intercept tests, we assessed pleiotropy bias, and Cochran’s Q statistics were used to evaluate heterogeneity to determine consistency among different IVs. With significant sensitivity analysis results, we could assess the robustness of primary analysis results, ensuring the research conclusions’ reliability without bias from potential statistical errors or data issues. Overall, our MR methods, IV selection and quality control, and sensitivity analysis followed MR analysis standard guidelines, providing deep insights into the causal effects between TL and diabetes and its complications. These findings not only enhance our understanding of the causal relationship between TL and diabetes and its complications, but also provide a solid scientific foundation for future prevention and treatment strategies.

This study systematically evaluated the bidirectional causal relationship between TL and diabetes and its complications using bi-directional MR methods, offering new perspectives and important scientific evidence for understanding diabetes’s pathological mechanisms. Although the overall analysis did not show a significant causal effect of TL on diabetes, subtype and complication analyses revealed the importance of TL in specific types of diabetes and its complications. These findings have significant clinical implications. First, TL can serve as a potential biomarker for assessing diabetes and complication risk, particularly in widely defined T1D and undefined diabetes [27,93,94,95]. Second, interventions targeting TL might play a role in preventing and treating diabetes and its complications [25,96,97]. For example, lifestyle improvements and proper glycemic control measures might help maintain telomere stability, reducing the risk of diabetes and its complications.

Despite utilizing large-scale GWAS data and bi-directional MR methods with high statistical power and causal inference capabilities, this study has some limitations. First, MR analysis depends on IV selection, and despite our efforts to choose appropriate SNPs, further validation is needed to confirm these SNPs fully meet IV assumptions. Second, there might be population differences in the definition and classification of diabetes and its complications, potentially affecting result accuracy. Finally, TL is influenced by various genetic and environmental factors, and this study could not entirely rule out all potential confounders. Furthermore, we must acknowledge that TL can be significantly affected by the growing use of anti-aging interventions, including various bioactive compounds and supplements, which represents a significant but often overlooked variable in current research. Melatonin supplementation, for example, has demonstrated considerable effects on cellular aging processes and telomere biology through its antioxidant and anti-inflammatory properties [98]. Studies have shown that such compounds can significantly modulate telomere dynamics and potentially counteract age-related cellular deterioration in experimental models [99,100]. This widespread but frequently undocumented use of anti-aging substances constitutes a potential source of hidden bias in our MR analysis, as genetic instruments may not fully capture these exogenous influences on TL. The consumption patterns of these bioactive compounds could vary systematically between diabetic and non-diabetic populations, further complicating the assessment of causality. To further validate and expand our findings, future research should consider the following areas. First, large-scale multicenter cohort studies integrating more genetic and environmental factors should be conducted to explore the complex relationship between TL and diabetes and its complications. Second, functional studies should reveal the specific biological mechanisms of telomere shortening in diabetes development and the impact of diabetes on telomere dynamics. Additionally, specific interventions for different diabetes types and complications should be explored, aiming to improve patient outcomes by delaying telomere shortening. In conclusion, this study provides new evidence of the bidirectional causal relationship between TL and diabetes and its complications, revealing their complex interactions. These findings not only offer new perspectives and scientific evidence for diabetes prevention and treatment, but also provide important directions for future research. Further studies are expected to help us better understand TL’s role in diabetes and its complications, leading to more effective intervention strategies and improved health outcomes for diabetes patients.

## 5. Conclusions

The bidirectional MR analysis confirmed that shortened TL significantly increases the risk of T1D and specific complications, particularly diabetic nephropathy and ketoacidosis. Importantly, our reverse MR analysis revealed a positive causal effect of T1D on TL, suggesting a compensatory mechanism activated in response to metabolic dysregulation. The elimination of these associations after adjusting for insulin therapy in multivariable MR highlights insulin’s critical role in regulating telomere homeostasis. These findings not only establish TL as a potential risk predictor for T1D and its complications, but also provide novel insights into the complex interplay between metabolic regulation, insulin signaling, and telomere biology in diabetes pathogenesis. Future research exploring these molecular pathways may lead to innovative therapeutic strategies targeting telomere maintenance in diabetes management.

## Figures and Tables

**Table 1:** The significant results derived from inverse MR analysis.

Method	*N*_snp_	*B*	*SE*	*p*-Value	Adjusting Insulin *
Type1 diabetes, definitions combined to TL					
MR Egger	14	0.0081	0.002	3.88 × 10^−^³	
Weighted median	14	0.0075	0.002	3.28 × 10^−5^	
IVW	14	0.0064	0.002	2.42 × 10^−5^	2.63 × 10^−1^
Simple mode	14	0.0065	0.004	1.48 × 10^−1^	
Weighted mode	14	0.0072	0.002	1.72 × 10^−3^	
Type 1 diabetes, strict (exclude DM2) to TL					
MR Egger	17	0.0077	0.002	2.67 × 10^−3^	
Weighted median	17	0.0073	0.002	3.95 × 10^−5^	
IVW	17	0.0060	0.001	4.08 × 10^−5^	2.77 × 10^−1^
Simple mode	17	0.0066	0.004	1.23 × 10^−1^	
Weighted mode	17	0.0072	0.002	1.12 × 10^−3^	
Type 1 diabetes without complications to TL					
MR Egger	31	0.0066	0.002	1.53 × 10^−3^	
Weighted median	31	0.0059	0.002	6.27 × 10^−4^	
IVW	31	0.0051	0.001	2.64 × 10^−4^	2.90 × 10^−1^
Simple mode	31	0.0064	0.005	1.91 × 10^−1^	
Weighted mode	31	0.0056	0.002	1.95 × 10^−3^	
Diabetic hypoglycemia to TL					
MR Egger	16	0.0046	0.005	3.49 × 10^−1^	
Weighted median	16	0.0061	0.004	9.10 × 10^−2^	
IVW	16	0.0055	0.003	4.16 × 10^−2^	9.78 × 10^−1^
Simple mode	16	0.0059	0.006	3.73 × 10^−1^	
Weighted mode	16	0.0059	0.004	1.19 × 10^−1^	
Diabetic retinopathy to TL					
MR Egger	16	−0.0248	0.012	5.77 × 10^−2^	
Weighted median	16	−0.0132	0.008	8.17 × 10^−2^	
IVW	16	−0.0113	0.006	4.19 × 10^−2^	7.34 × 10^−1^
Simple mode	16	−0.0002	0.013	9.87 × 10^−1^	
Weighted mode	16	−0.0164	0.008	5.63 × 10^−2^	
Type 1 diabetes with coma to TL					
MR Egger	16	0.0031	0.001	5.62 × 10^−2^	
Weighted median	16	0.0026	0.001	6.60 × 10^−2^	
IVW	16	0.0025	0.001	2.54 × 10^−2^	4.09 × 10^−1^
Simple mode	16	0.0047	0.002	6.97 × 10^−2^	
Weighted mode	16	0.0025	0.001	9.32 × 10^−2^	

* Multivariate Mendelian randomization analysis adjusted for the use of insulin (ukb-b-15445) to obtain *p*-values from the inverse-variance weighted method.

## Data Availability

The datasets generated and/or analyzed during the current study are available in the IEU open GWAS project (https://gwas.mrcieu.ac.uk/, accessed on 20 December 2024) and FinnGen database (https://risteys.finngen.fi/, accessed on 20 December 2024).

## Supplementary Materials

The following supporting information can be downloaded at: https://www.mdpi.com/article/10.3390/biomedicines13040774/s1, Table S1. All datasets used in the MR analysis; Table S2. IVs related to telomere length with R2 and F values; Table S3. All results of forward MR analysis; Table S4. Heterogeneity and pleiotropy of forward MR analysis; Table S5. All results of inverse MR analysis; Table S6. Sensitivity analysis for inverse MR analysis; Figure S1. Causal effect of singe SNP for forward MR analysis; Figure S2. Leave-one-out analysis for forward MR analysis. References [39,101] are cited in the Supplementary Materials.
