# Supplementary figures and images for: Telomere Length as Both Cause and Consequence in Type 1 Diabetes: Evidence from Bidirectional Mendelian Randomization
Source: Biomedicines. 2025 Mar 22;13(4):774. doi: 10.3390/biomedicines13040774 (PMC12024553; doi:10.3390/biomedicines13040774)

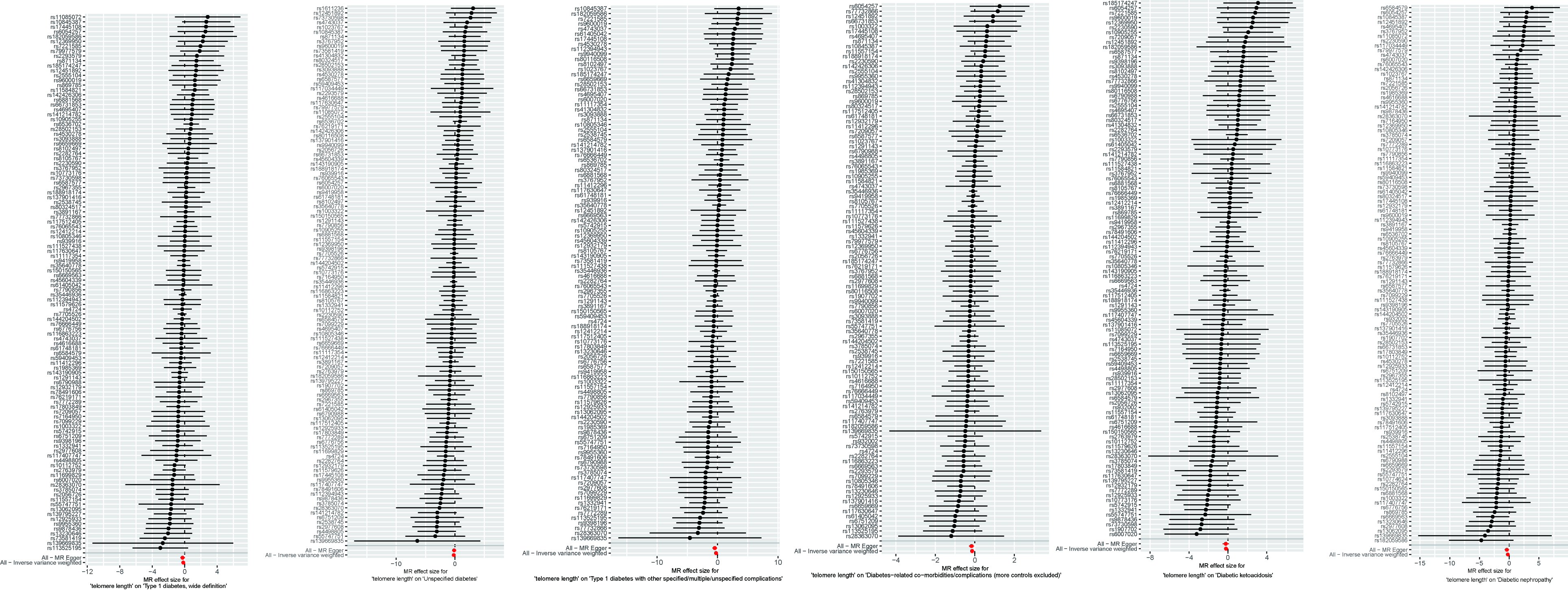

Supplement: Supplementary file 1 [file biomedicines-13-00774-s001.zip › Figure S1 Causal effect of singe SNP for forward MR analysis .tif]

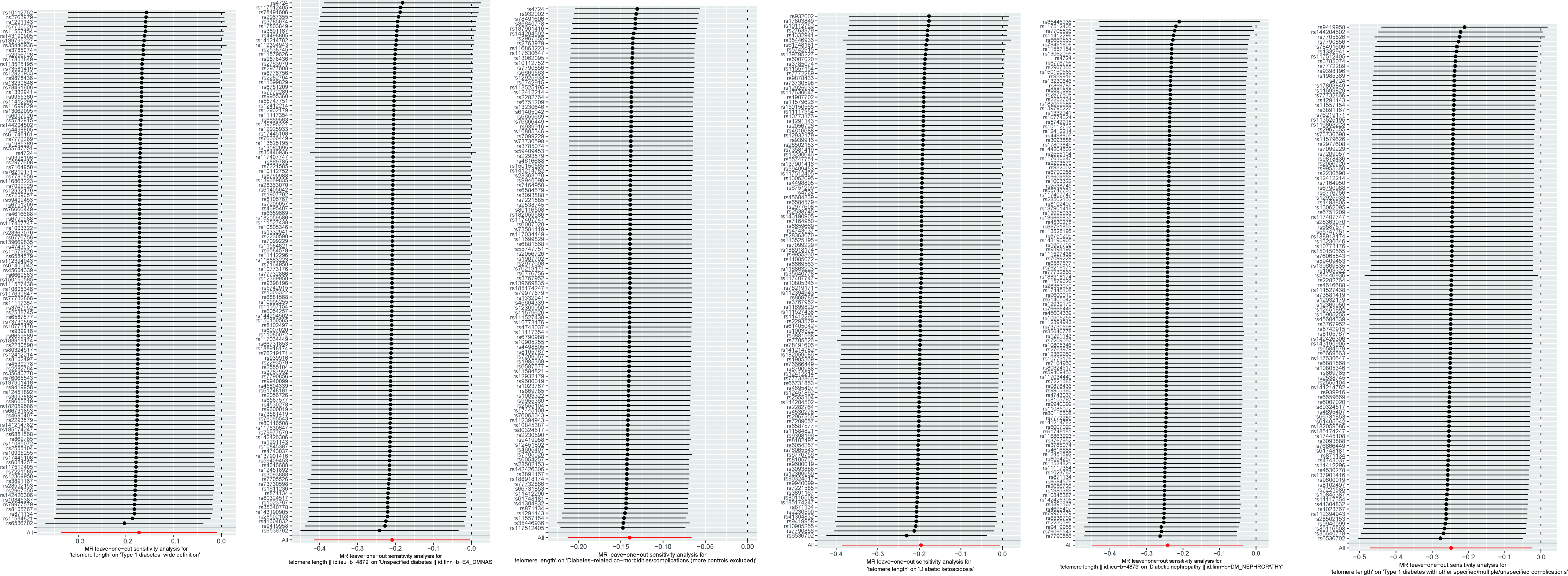

Supplement: Supplementary file 1 [file biomedicines-13-00774-s001.zip › Figure S2 Leave-one-out analysis for forward MR analysis.tif]
